# Lactic Acid Bacteria of Marine Origin as a Tool for Successful Shellfish Farming and Adaptation to Climate Change Conditions

**DOI:** 10.3390/foods13071042

**Published:** 2024-03-28

**Authors:** Iva Čanak, Deni Kostelac, Željko Jakopović, Ksenija Markov, Jadranka Frece

**Affiliations:** Faculty of Food Technology and Biotechnology, University of Zagreb, Pierottijeva 6, 10 000 Zagreb, Croatia; icanak@pbf.hr (I.Č.); deni.kostelac@pbf.unizg.hr (D.K.); kmarkov2@pbf.hr (K.M.)

**Keywords:** climate change, shellfish, marine origin lactic acid bacteria, probiotics

## Abstract

Climate change, especially in the form of temperature increase and sea acidification, poses a serious challenge to the sustainability of aquaculture and shellfish farming. In this context, lactic acid bacteria (LAB) of marine origin have attracted attention due to their ability to improve water quality, stimulate the growth and immunity of organisms, and reduce the impact of stress caused by environmental changes. Through a review of relevant research, this paper summarizes previous knowledge on this group of bacteria, their application as protective probiotic cultures in mollusks, and also highlights their potential in reducing the negative impacts of climate change during shellfish farming. Furthermore, opportunities for further research and implementation of LAB as a sustainable and effective solution for adapting mariculture to changing climate conditions were identified.

## 1. Introduction

Mariculture has emerged as a significant industry globally, providing a sustainable source of seafood to meet the rising demand. From major species to regional production, the sector continues to expand and diversify. However, environmental challenges persist, necessitating a focus on sustainability and responsible practices [1,2,3]. By addressing these challenges, mariculture can continue to thrive, contributing to food security and economic development while safeguarding natural resources and protecting biodiversity. Climate change stands as one of the most pressing challenges of our era, and its repercussions are reverberating across diverse sectors, including aquaculture. As the Earth’s climate continues to warm, the mariculture industry faces a range of impacts that can influence production, species distribution, and overall sustainability. One of the primary concerns of climate change on aquaculture is the alteration of water temperatures. As global temperatures rise, water temperatures in rivers, lakes, and oceans also increase. This poses challenges for aquaculture operators as different species have specific temperature requirements for optimal growth and reproduction. Higher temperatures can lead to reduced growth rates, decreased feed conversion efficiency, and increased susceptibility to diseases in certain species [1,4].

Climate change brings about changes in precipitation patterns and a heightened occurrence of extreme weather events, both of which directly impact aquaculture operations. Heavy rainfall and flooding can result in the release of excess nutrients and pollutants to aquatic systems, potentially leading to poor water quality and increased disease outbreaks. Conversely, droughts and reduced freshwater availability can constrain aquaculture production, particularly in areas that rely on freshwater sources for fish farms or land-based facilities.

Sea-level rise is another significant concern for coastal aquaculture operations. As oceans warm and expand, combined with the melting of ice sheets and glaciers, sea levels are projected to rise. This can lead to the inundation of coastal fish farms and the intrusion of saltwater into freshwater systems, affecting the growth and survival of cultured species that are sensitive to salinity changes [5,6].

However, the mariculture industry is not passive in the face of these challenges. Innovative adaptation and mitigation strategies are being developed to minimize the impacts of climate change on mariculture [7,8,9,10,11,12].

**Figure 1:**
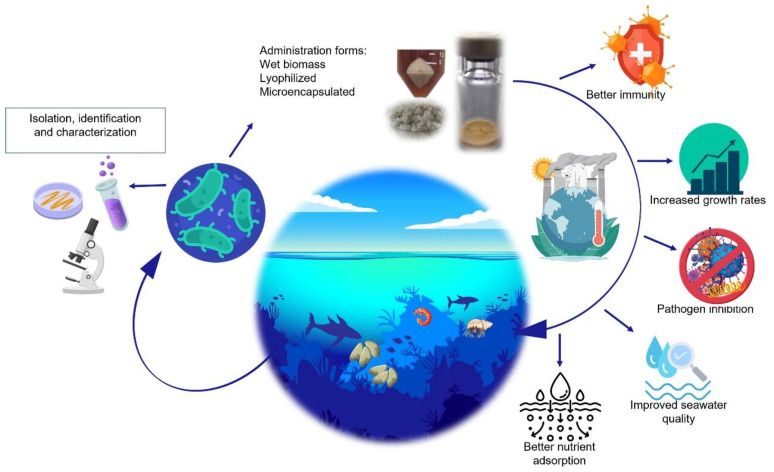
Use of lactic acid bacteria as toll for adaptation to climate change.

As an alternative to chemotherapy and vaccination, probiotics have been increasingly employed in aquaculture to manage the emergence of various diseases and new strains of pathogenic microorganisms resulting from rising temperatures linked to climate change (Figure 1).

The use of lactic acid bacteria (LAB) isolated from the marine environment offers several advantages, including better adaptation and higher efficiency. This becomes particularly important due to factors such as the emergence of new aquatic species, climate change, and consumer demands for healthy, natural, and chemical-free food options. As a result, alternative methods are being sought, and one such strategy is the use of natural bacterial biological preservatives. Derived from LAB, these preservatives are deemed safe for consumption as they are inherent components of the natural beneficial microbiota present in the human diet.

Considering these factors, it is evident that LAB derived from marine sources have significant potential for applications in mariculture. They can serve as probiotics, providing a protective role, or as bio preservatives, enabling the production of innovative and healthy products with an extended shelf life. The purpose of this work is to consolidate previous research on marine-origin LAB in order to emphasize their protective role in shellfish cultivation and to showcase their potential and possibilities in cultivating shellfish amidst changing climate conditions.

## 2. Lactic Acid Bacteria

Lactic acid bacteria (LAB) are a natural part of the microbiota found in the digestive system of humans and animals. They possess various beneficial properties that contribute to maintaining intestinal balance and influencing the biological processes of the host. LAB are non-sporulating, gram-positive, microaerophilic, non-motile, and catalase-negative microorganisms. Their key characteristic is the ability to produce lactic acid through the fermentation of carbohydrates.

LAB can be classified into two groups based on the final fermentation product: homofermentative and heterofermentative. The homofermentative group primarily produces lactic acid as the end product of glucose fermentation. On the other hand, the heterofermentative group also generates carbon dioxide, acetic acid, and ethanol in addition to lactic acid.

Various species of LAB exist within different genera, including *Lactobacillus*, *Lactococcus*, *Oenococcus*, *Streptococcus*, *Enterococcus*, *Pediococcus*, *Vagococcus*, *Tetragenococcus*, *Leuconostoc*, *Carnobacterium*, *Sporolactobacillus*, and *Weissella*. With the exception of the genus *Bifidobacterium*, LAB generally have low G + C content.

Throughout their evolutionary journey, LAB have undergone genetic changes resulting in the loss of genes responsible for cofactor synthesis and the biosynthetic pathways for certain amino acids and vitamins. Consequently, LAB require nutrient-rich media for their growth and development [13].

LAB have an antimicrobial effect, which is manifested in the ability to produce antimicrobial substances. Antimicrobial substances of LAB can be divided into the following two groups: compounds of low molecular mass (mass up to 1000 Da) and compounds of high molecular mass (mass greater than 1000 Da) [14].

The group of low molecular weight compounds includes organic acids, hydrogen peroxide, diacetyl, acetaldehyde, acetoin, carbon dioxide, reuterin, reutericycline, and others. The most important organic acids with antimicrobial activity are lactic and acetic acid. The inhibitory effect is most often caused by the undissociated form of the organic acid, which diffuses through the cell membrane towards the more alkaline cytosol, where it affects the functions of essential metabolites. The toxic effects of lactic and acetic acid include a decrease in the intracellular pH value and disruption of the membrane potential. The antimicrobial activity of hydrogen peroxide consists of the ability to oxidize bacterial cells and destroy the molecular structures of cellular proteins. Diacetyl, acetaldehyde, and acetoin are products of heterofermentative LAB. Diacetyl and acetaldehyde are present in fermented milk products where they regulate the growth of contaminants, which is why they are used as preservatives. The antimicrobial activity of carbon dioxide is manifested through the creation of anaerobic conditions, the inhibition of enzymatic decarboxylation, and the influence on membrane permeability due to the ability to accumulate in the lipid bilayer. Reuterin and reutericycline are products of the bacterium *Lactobacillus reuteri*. Reutericycline inhibits the growth of gram-positive and gram-negative bacteria, fungi and protozoa, while reuterin inhibits the growth of gram-positive bacteria [14].

Bacteriocins, particularly notable among antimicrobial substances of high molecular mass, represent a significant class. Produced by LAB, these compounds are peptides or proteins synthesized within ribosomes, exhibiting antimicrobial properties primarily against other gram-positive bacteria. Among LAB bacteriocins, nisin stands out as a prominent example, recognized as the sole approved preservative in this category [14].

Benefiting from their Generally Recognized As Safe (GRAS) status, LAB are used extensively within the fermented food industry, enriching products with enhanced taste, texture, and nutritional profiles. Beyond food production, LAB hold promise in biorefineries, contributing to the synthesis of various valuable compounds like lactic acid, polyols, and vitamins [15].

The sequencing of over 75 LAB genomes has markedly enhanced our comprehension of their traits and mechanisms of action. This genomic insight facilitates industrial processes and the development of genetic engineering techniques, aiming to tailor LAB strains with specific and desirable traits [15].

### Marine Origin Lactic Acid Bacteria

Although LAB are a very well-studied and characterized group of bacteria, few studies have been published on those of marine origin. What is less known is the importance of marine origin LAB in the decomposition of organic substances and in the food and pharmaceutical industry [16].

Deep sea sediments contain a large amount of bacteria from the genera Firmicutes, Cytophaga, Spirochaetes, and Proteobacteria.

Research conducted by Sica et al. [17] with samples isolated from fish species of the Bahía Blanca estuary monitored the competing properties of strains from the genera *Weissella*, *Lactobacillus*, *Enterococcus*, *Leuconostoc*, and *Pedicoccus*. LAB strains were tested against the pathogen *Listeria monocytogenes* as an indicator microorganism, given that fish and fish products are frequent carriers of this pathogen and it is often isolated from them, but also against the pathogens *Aeromonas salmonicida*, *Yersinia ruckeri*, and *Lactococcus garvieae*. The obtained results showed a certain degree of competing properties of all isolated LAB strains against the mentioned pathogens, which points to their potential for use as probiotics and biopreservatives in mariculture.

Furthermore, Muñoz-Atienza et al. [18] conducted a study to evaluate the antimicrobial and bactericidal properties of LAB isolates obtained from marine animals commonly consumed by humans. Among the 99 strains tested in vitro, 33 showed promising results (including *Enterococcus faecium*, *Pediococcus pentosaceus*, *Lactobacillus sakei* subsp. *carnosus*, *Lactobacillus curvatus* subsp. *curvatus*, *Lactococcus lactis* subsp. *cremoris*, *Leuconostoc mesenteroides* subsp. *cremoris*, and *Weissella cibaria*). These strains hold potential for use as probiotics in mariculture and for mitigating pathogen spread in marine ecosystems. However, further in vivo research is necessary to unequivocally establish the safety of these LAB strains for practical application.

In their research, Čanak et al. [19] characterized *Lactiplantibacillus plantarum* O1, previously isolated from sea bream (*Sparus aurata*). The results showed good survival in a wide pH and temperature range and strong competing properties as a result of the production of plantaricin A. As a follow-up to this research, survival tests were also conducted in the in vitro conditions of the gastrointestinal tract of fish and seawater, where the strain *L. plantarum* O1 showed a high percentage of survival and potential for application in mariculture [20].

However, it is also known that some marine origin LAB have a bad effect on marine animals, for example the pathogenic bacterium *Streptococcus iniae*, which is found in both fresh and salt water, and was originally isolated from the subcutaneous abscesses of Amazonian river dolphins. *S. iniae* is one of the most serious pathogens of fish aquaculture with a mortality rate of 30–50%. Also, some other LAB pathogenic strains, such as *Carnobacterium piscicola*, *Lactococcus garvieae*, and *Lactococcus piscium*, have been reported as occasional causes of fish mortality [16].

*Lactic acid bacteria isolated from shellfish*

Due to their previously mentioned method of nutrition, i.e., filtering water through the body, many cultures of microbes are found in bivalves, which can be divided into indigenous and non-indigenous microorganisms. The microbiota of bivalves reflects the microbial population of the water in which they grow, among which various species and genera of LAB can be found.

The gastrointestinal (GI) tract microbiota of shellfish can be divided into two main groups: the GI lumen microbiota (allochthonous microbiota) and the mucosal surface adherent microbiota (indigenous microbiota). While many studies have focused on examining both types of microbiota obtained from the entire intestine, there has been less emphasis on understanding the indigenous gut microbiota.

According to Merrifield et al. [21], shellfish harbor several species of indigenous bacteria including *Lactobacillus*, *Lactococcus*, *Leuconostoc*, *Enterococcus*, *Streptococcus*, *Carnobacterium*, *Pediococcus*, and *Weissella*. Although there are fewer studies on indigenous gut bacteria in shellfish compared to finfish, LAB have consistently been found in the GI tracts of various shellfish species such as shrimp, prawns, swimming crab, mud crab, and different mollusks.

It has been scientifically proven that certain strains have an inhibitory effect and competing properties against some bacteria. Pinto et al. [22] noted that *Enterococcus faecium* and *Pediococcus pentosaceus* species isolated from fresh oysters and mussels possess competing properties against *Listeria monocytogenes* bacteria.

In 2020, Pavlova et al. [23] undertook a comprehensive investigation into the antifungal capabilities of LAB derived from the Mediterranean mussel (*Mytilus galloprovincialis*). The study aimed to tackle the contamination of mussels by various pathogenic microorganisms, such as *Escherichia coli*, *Staphylococcus aureus*, *Salmonella typhimurium*, and *Vibrio cholera*, which are linked to sea pollution stemming from tourism and construction activities along the Black Sea coast.

Through their research, two LAB isolates were identified as *Lactobacillus* sp., exhibiting notable antifungal properties. The study unveiled several compounds contributing to this activity, encompassing organic acids, low molecular weight compounds, phenylacetic acid, fatty acids, cyclic dipeptides, protein compounds, and various other substances like lactones. These findings led to the conclusion that LAB isolated from mussels could serve as a bioprotective culture to hinder the proliferation of pathogens.

In 2010, Lee et al. [24] undertook the isolation of *Lactobacillus* spp. from sea oysters (*Crassostrea gigas*), targeting strains resilient to external stressors and environmental influences. A total of 83 lactobacilli strains were successfully isolated. To assess their efficacy against pathogens, the selected strains underwent in vitro testing, measuring the diameter of inhibition zones. Among these strains, *Lactobacillus rhamnosus* exhibited the largest inhibition zone diameter, indicating strong antagonistic activity against pathogens. Consequently, it was identified as the most resilient strain and deemed highly promising for use as a food supplement in marine aquaculture.

The goal of the research conducted in 2014 by Fajardo et al. [25] was to isolate bacteria with potentially probiotic properties from shellfish, which could also facilitate the purification of mollusks for commercial use. A comprehensive study involved the isolation of 365 bacteria from the digestive glands of bivalves, with a subsequent investigation into their activity against diverse pathogens. Among these isolates, strain 3M21, identified as the LAB strain *Enterococcus hirae*, demonstrated remarkable effectiveness. It exhibited activity against *Listeria monocytogenes*, *Listeria innocua*, and *Enterococcus faecalis*. Additionally, it displayed activity against hepatitis A and mouse norovirus. Notably, this strain also produces an active substance identified as a bacteriocin, further enhancing its antimicrobial properties.

## 3. Probiotics

Given that some antibiotics used in aquaculture are also used to treat bacterial infections in humans, bacterial strains that become resistant to antibiotics appear, resulting in an increase in mortality from some diseases. Due to all of the above, non-pathogenic microorganisms, especially probiotics, have started to be used as an alternative in mariculture, with the aim of preserving the health of marine animals. Probiotics are microorganisms that inhibit the growth of pathogenic microorganisms in different ways; for example, they modify the intestinal microbiota, strengthen the immune system and prevent the growth of pathogenic microorganisms by excreting organic acids, bacteriocins, enzymes, and hydrogen peroxide [14,26,27].

Probiotic preparations can contain one or more selected microbial strains, and those for human use are most often LAB belonging to the genera *Lactobacillus*, *Bifidobacterium*, and *Lactococcus*, *Streptococcus*, *Enterococcus* [28]. If applied for human and animal use, probiotics should have GRAS (Generally Recognized as Safe) status regulated by the FDA in the USA, or QPS status (Qualified Presumption of Safety), regulated by the EFSA in Europe [29].

The utilization of terrestrial bacterial strains as probiotics in mariculture encounters limited success due to strains’ characteristics being closely tied to their original environment. Consequently, the pursuit of probiotic bacteria from marine habitats presents a more promising approach, as these strains are anticipated to exhibit greater efficacy in aquaculture applications compared to their terrestrial counterparts.

Marine animals serve as prevalent reservoirs of lactic acid bacteria capable of producing bacteriocins. Moreover, reports indicate the isolation of such strains from diverse marine sources including sea soil, sediment, and seaweed, underscoring the vast diversity of bacteriocin-producing strains within the marine ecosystem.

**Figure 2:**
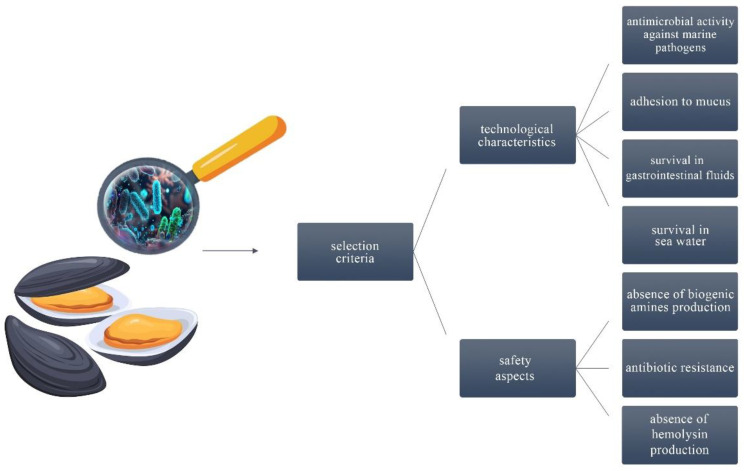
Selection criteria for marine origin probiotics [20,30].

Criteria for the selection of such strains are illustrated in Figure 2, delineating the key factors considered in the process.

It has been noted that LAB employed as probiotics demonstrate the capability to impede the growth of marine pathogens such as *Aeromonas salmonicida* and *Vibrio anguillarum*, thereby mitigating the incidence of fish diseases. While most LAB species hold GRAS and Qualified Presumption of Safety (QPS) statuses, only the strain *Pediococcus acidilactici* MA 18/5M has gained approval as a probiotic for application in aquaculture within European Union countries [26].

In recent years, numerous LAB strains have been isolated from marine-derived products including cold-smoked salmon, oysters, shellfish, Atlantic cod, and Mediterranean fish [31].

Moreover, investigations conducted on mud crabs (*Scylla paramamosain*), commercially significant in southeastern China, unveiled that LAB strains such as *E. faecalis* and *P. pentosaceus*, extracted from crab intestines, exhibit inhibitory effects against major pathogens affecting this species, including *Aeromonas hydrophila*, *Vibrio parahaemolyticus*, *Vibrio alginolyticus*, *Staphylococcus aureus*, and group B streptococci. Notably, these LAB strains display a wide tolerance range to varying pH levels (2–10) [32].

According to the obtained results, the authors concluded that the mentioned LAB strains can be used as probiotics in the future because they showed improved immunity, faster growth of cancer, and increased protection of the intestinal system [32].

## 4. Application of Probiotics in Sustainable Cultivation of Shellfish

To ensure the prevention and control of shellfish diseases, various measures have been implemented. One important aspect of shellfish disease prevention is the use of biosecurity measures. Biosecurity measures are designed to thwart the introduction and dissemination of pathogens within shellfish mariculture systems. These measures include strict hygiene practices, monitoring and testing for pathogens, quarantine procedures for incoming shellfish stocks, and the use of disinfectants and sterilization techniques for equipment and facilities. Another important aspect of shellfish disease prevention is the promotion of healthy and robust shellfish populations. This can be achieved through good husbandry practices, such as proper nutrition, optimal water quality management, and regular health monitoring. In addition, the use of vaccination and immunostimulation strategies has shown great potential in preventing shellfish diseases. According to a study by Labh and Shakya [33], vaccination can enhance the immune response of shellfish, improving their resistance to diseases. Furthermore, understanding the ecology of shellfish diseases is crucial for their prevention. This includes studying the interactions between pathogens, shellfish hosts, and environmental factors. By identifying the key factors influencing disease outbreaks, such as water temperature, salinity, and nutrient concentrations, aquaculturists can implement targeted management strategies to mitigate the risk of disease transmission [34,35].

Recently, emphasis has been placed on examining the impact of the addition of probiotic cultures on the growth and development of shellfish in order to prevent disease. A large number of tests so far have already confirmed the positive effect of probiotic supplementation on the health of marine animals.

Research exploring the efficacy of probiotics on molluscan shellfish is comparatively limited in comparison to crustacean shellfish. Nonetheless, abalone and oysters stand out as the most extensively studied molluscan species concerning probiotic applications.

A diverse range of probiotics, including *Bacillus*, *Lactobacillus*, and *Enterococcus*, have undergone testing in the mariculture of various abalone species. In most of these trials, probiotics were administered through diet, yielding several positive outcomes. These include heightened immunity, increased expression of immune-related genes, enhanced activity of digestive enzymes, and improvements in growth and survival rates.

Studies examining the impact of probiotics on oysters have been somewhat limited, predominantly concentrating on growth performance, survival rates, and resistance to pathogens. Aguilar-Macías et al. [36] discovered that juvenile pearl oysters (*Pinctada mazatlanica*) exhibited significantly enhanced shell length, weight gain, and survival rates when fed with probiotic-supplemented diets compared to those without probiotics. Similar positive effects were observed in Cortez oysters (*Crassostrea corteziensis*) after being fed diets enriched with probiotics, as reported by Campa-Córdova et al. [37].

In a study conducted by Sohn et al. [38], the introduction of probiotic strains, *Phaeobacter inhibens* and *Bacillus pumilus*, into the rearing water of Eastern oyster (*Crassostrea virginica*) larvae demonstrated increased protection against specific pathogens. These findings underline the potential of probiotics to enhance the health and resilience of oyster populations in aquaculture settings.

However, research dealing with the impact of probiotic supplementation on the growth of bivalve mollusks in conditions of climate change is rather lacking. Kovačić et al. [11] and Čanak et al. [12] successfully demonstrated the positive effect of the addition of the indigenous probiotic culture *Lactiplantibacillus plantarum* I on the health status of queen scallop under natural conditions as well as under simulated conditions of climate change. The LAB isolate exhibited competing properties, effectively inhibiting disease-causing microorganisms. Additionally, an increase in both the growth rate and weight, as well as the growth rate length, of the queen scallop was observed.

However, additional research is needed, as well as the isolation of new marine indigenous LAB isolates to better understand the mutual interactions between shellfish and microorganisms and to use them as a tool for successful adaptation to climate change conditions.

*Probiotics in the prevention of shellfish disease*

Specific bacterial pathogens represent a significant threat to fish and shellfish populations, particularly in intensive breeding environments where natural immunity may decline. To combat this issue, laboratories and hatcheries have historically relied on methods such as water and food disinfection and the prophylactic or therapeutic use of antibiotics to bolster host resistance. However, the widespread use of antibiotics in disease control within the fish industry has spurred the emergence of antibiotic-resistant pathogens, with the potential for horizontal gene transfer exacerbating this concern [39].

An alternative strategy to mitigate opportunistic infections by fish pathogens involves the manipulation of intestinal microbiota through the introduction of antagonistic bacteria into the diet. This approach aims to enhance the proportion of beneficial bacteria within the intestinal microbiota [40]. A notable advantage of this method is its applicability during the early stages of development, when vaccination may be impractical due to an underdeveloped immune system. In this context, the sustained competing properties exhibited by probiotic LAB strains emerge as a crucial characteristic.

Limited research has been conducted on the effectiveness of probiotics in molluscan compared to crustacean. However, abalone and oyster species were the most studied. Various probiotics, encompassing strains of *Bacillus*, *Lactobacillus*, *Exiguobacterium*, *Vibrio*, and *Enterococcus*, have been subjected to testing in the aquaculture of diverse abalone species. In most trials, probiotics were administered through diet, yielding numerous favorable outcomes. These include bolstered immunity, heightened expression of immune-related genes, increased activity of digestive enzymes, and enhancements in both growth and survival rates [41].

A study conducted by Gao et al. [42] illustrated the beneficial effects of incorporating indigenous *Lactobacillus pentosus* into the diet of abalone (*Haliotis discus hannai* Ino) over an eight-week period. This intervention led to significantly improved immune responses, enhanced feed efficiency, elevated survival rates, and reduced mortality during a challenge test with *V. parahaemolyticus.*

Likewise, the utilization of multi-strain probiotics, comprising *Exiguobacterium* JHEb1, *Vibrio* JH1, and *Enterococcus* JHLDc, incorporated into the diet of New Zealand abalone (*Haliotis iris*), has yielded various beneficial effects. Studies by Hadi et al. [43], Grandiosa et al. [44], and Grandiosa et al. [45] reported improvements in shell length, wet weight, immune response, and resistance against *V. splendidus*.

In contrast, research on the effects of probiotics on oysters has received relatively scant attention, with most investigations focusing on growth performance, survival rates, or pathogen resistance. Studies conducted on pearl oysters (*Pinctada mazatlanica*) and Cortez oysters revealed enhancements in shell length, weight gain, and survival rates upon supplementation with probiotics. Meanwhile, in the case of Eastern oyster larvae, probiotics administered in rearing water conferred protection against specific pathogens, although their impact on growth and survival remained inconclusive [37,38].

Some probiotic candidates exhibit promising potential in bolstering resistance against challenges with *Vibrio* spp. and other pathogens in oyster larviculture, although further research is imperative to fully grasp their capabilities [46].

Additionally, certain commercial aquaculture products have integrated prebiotics into their formulations, such as mannan, glucan, and yucca extract, to augment the beneficial effects of these products [47].

Currently, commercial probiotic preparations are accessible in liquid or powder form. In recent years, systems have been developed for the immobilization of probiotics, particularly through microencapsulation. This technique involves encapsulating high-density microbial cells within a colloidal matrix using substances like alginate, chitosan, carboxymethylcellulose, or pectin, providing physical and chemical protection. Although lyophilized commercial preparations offer advantages in terms of storage and transport, proper reconstitution conditions, including temperature, degree of hydration, and solution osmolarity, which are vital to ensure the viability of the bacteria [48].

The advantages of using probiotics in mariculture and their effectiveness are outlined below.

### 4.1. Growth Promoters

Probiotics are employed in aquaculture to promote the growth of cultured species, yet their mechanism of action regarding appetite enhancement or digestibility improvement remains incompletely understood [49]. Studies have shown that supplementing the diet of Nile tilapia (*Oreochromis niloticus*) with a probiotic *Streptococcus* strain resulted in significant increases in protein and lipid content, as well as overall fish weight [50]. Likewise, the growth and survival of aquarium fishes, such as wrasse (*Xiphophorus helleri*) and guppy (*Poecilia reticulate*), were notably enhanced when they were fed diets supplemented with *B. subtilis* and *Streptomyces* [51,52].

Probiotics have also demonstrated efficacy in shellfish. For instance, the growth rate of Peter’s small and large ears improved by 8% and 34%, respectively, over an eight-month period when supplemented with probiotics. Additionally, a diet supplemented with probiotics led to a 62% survival rate in bivalves infected with the pathogenic bacterium *Vibrio anguillarum*, compared to only 25% survival in untreated bivalves [53]. Similar positive outcomes were observed in the research conducted by Čanak et al. [12], where queen scallops fed with the addition of *Lpb. plantarum* I exhibited increased weight and length compared to those in control tanks fed only with live algae mix culture.

### 4.2. Digestion of Nutrients

Balcázar et al. [54] concluded that probiotics exert a positive influence on the digestive tract of marine animals due to their ability to synthesize proteases, amylases, and lipases, along with vitamins, fatty acids, and amino acids. Consequently, the supplementation of food with probiotics enhances nutrient absorption efficiency [55]. In white shrimp and Indian white shrimp (*Fenneropenaeus indicus*), the incorporation of probiotics led to increased digestibility of dry matter, protein, and phosphorus [56]. Chai et al. [57] discovered that *Bacillus* strains isolated from healthy, wild shrimps bolstered Pacific white shrimp growth and improved feed conversion, digestion, and nutrient absorption. Similarly, Zhao et al. [58] observed enhanced nutrient status in abalone fed with *B. stratosphericus*, corroborating these findings.

In their review paper, Ringø et al. [30] also stated that some of the current research recorded a positive effect on the breakdown of nutrients in terms of increasing enzyme activity (protease, amylase, and lipase) by adding probiotic strains to the shellfish diet.

### 4.3. Seawater Quality

Fish farmers can mitigate the accumulation of dissolved organic carbon during the growing season by sustaining elevated levels of probiotics in ponds. Additionally, probiotics play a role in balancing phytoplankton production [54]. Lalloo et al. [59] isolated multiple *Bacillus* species from carp (*Cyprinus carpio*) and assessed their impact on water quality during aquarium fish cultivation, as well as their effect on the growth of *A. hydrophila* with the addition of nine strains. Three *Bacillus* isolates exhibited significant pathogen inhibition capability while simultaneously reducing concentrations of anorganic salts. Similarly, Wang et al. [60] demonstrated that a commercial preparation comprising *Bacillus* sp., *S. cerevisiae*, *Nitrosomonas* sp., and *Nitrobacter* sp. effectively enhanced the beneficial microbiota of white shrimp while decreasing levels of inorganic nitrogen and phosphate.

### 4.4. Stress Tolerance

Stress involves a number of physical and chemical factors that can cause illness or death. Aquatic animals encounter various stressors during cultivation, including transportation, inadequate nutrition, high stocking density, temperature fluctuations, oxygen deprivation, chemical exposure, pesticides, and changes in water salinity, and the cause of most of these stressors is climate change [61,62].

Castex et al. [63] proposed that the antioxidative properties of a *Lactobacillus fermentum* strain could serve as protective mechanisms within the intestinal microbial ecosystem, aiding in combating both exogenous and endogenous oxidative stresses. More recently, the addition of *Lb. plantarum* to the diet significantly enhanced stress resistance in shrimp subjected to acute low salinity [64]. Moreover, this dietary supplementation has been shown to enhance environmental adaptability, maintain redox balance, and stimulate immune function in abalone, additionally confirming the positive role of probiotics in adapting to the conditions of ecological valence [42].

Recent research has delved into understanding the mechanisms by which probiotics enhance immune function, spurred by numerous findings indicating their immunomodulatory effects. For instance, in tiger shrimp (*Penaeus monodon*), the inclusion of dietary *Bacillus* sp. S11 has been linked to improved cellular and humoral immunity, resulting in heightened disease resistance [65]. Similarly, the application of probiotic *Clostridium butyricum* CBG01 has demonstrated the ability to enhance the immunity of whiteleg shrimp (*Litopenaeus vannamei*). This enhancement is evidenced by significantly increased activities of alkaline phosphatase, acid phosphatase, total nitric oxide synthase, and lysozyme [66]. Moreover, various other immune parameters, including hemolymph bactericidal activity, phagocytic activity, superoxide dismutase (SOD) activity, total antioxidant capacity, total hemocyte count, catalase activity, phenoloxidase, and prophenoloxidase, have been reported to be positively influenced by probiotics in various shellfish species [67,68,69,70].

Furthermore, numerous studies have shown that the addition of probiotics to shellfish diets enhances the expression of immune-related genes, such as pen-3a, proPO, SOD, HSP70, and lipopolysaccharide and β-1,3-glucan binding protein (LGBP) [32,57,67,71,72,73,74,75,76,77].

## 5. Challenges in Shellfish Cultivation in Climate Change Conditions and Potential Solutions

Rising global average temperatures and changing precipitation patterns can directly impact the growth and survival of shellfish species. These challenges can have detrimental effects on shellfish cultivation, including reduced larval survival, delayed metamorphosis, and lowered growth during development [78,79] To mitigate the negative effects of climate change on shellfish cultivation, there are potential solutions that can be implemented.

- Implementing adaptive management strategies: Shellfish farmers can respond to shifting environmental conditions by consistently monitoring water quality, temperature, and pH levels. Using these data, they can adjust their practices such as feed management, stocking densities, and harvest timing to maximize growth and survival rates.
- Adopting sustainable aquaculture practices: Practices like integrated multi-trophic aquaculture systems offer a means to mitigate climate change risks in shellfish farming. These systems involve cultivating various species, like shellfish and seaweed, in tandem. This approach can reduce water nutrient levels, provide supplementary food for shellfish, and potentially counteract ocean acidification through adjacent seaweed photosynthesis.
- Developing resilient shellfish varieties: Research and breeding programs can concentrate on creating shellfish strains that are better equipped to handle changing environmental conditions. These varieties may possess genetic traits or be selectively bred for heightened tolerance to elevated temperatures, lower pH levels, and other climate change-related stressors.
- Implementing conservation and restoration efforts: safeguarding and reviving natural habitats such as seagrass beds, salt marshes, and oyster reefs can serve as natural defenses against climate change impacts on shellfish farming.
- Fostering collaboration between climate change scientists and aquaculture practitioners: by collaborating, these two groups can exchange knowledge and research outcomes and best practices to devise strategies and solutions addressing climate change challenges in shellfish farming.
- Investing in monitoring and research: Sustained research and monitoring endeavors are vital for a deeper understanding of different shellfish species’ vulnerabilities to climate change. This knowledge can guide the development and application of targeted adaptation strategies.
- Implementing policy and regulatory measures: governments and regulatory bodies hold a pivotal role in supporting shellfish farmers through policies fostering sustainable aquaculture practices, incentivizing climate change adaptation, and providing funding for research and infrastructure enhancements [80,81].

Probiotics, beneficial microorganisms that promote the health of their host organism, are emerging as a potential solution to enhance the resilience of shellfish in the face of climate change. The application of probiotics in shellfish farming involves introducing specific strains of bacteria to positively influence the microbiome of the shellfish and the surrounding water [30,82].

The positive effects of probiotic cultures are mentioned below:

(1) Enhanced immune function: Probiotics have the potential to boost the immune system of shellfish, making them more resistant to diseases that may become more prevalent as a result of changing environmental conditions. By fostering a healthy microbial community, probiotics help create a protective barrier against harmful pathogens.
(2) Improved nutrient utilization: Climate change can affect the availability and distribution of nutrients in the water. Probiotics play a role in enhancing the efficiency of nutrient utilization by shellfish, ensuring optimal growth even under suboptimal conditions.
(3) Mitigation of harmful algal blooms: Certain probiotic strains have shown promise in preventing or mitigating harmful algal blooms. By outcompeting harmful algae for nutrients or producing substances that inhibit their growth, probiotics can help maintain a balanced and healthy aquatic environment for shellfish [83].

The challenges posed by climate change to shellfish farming necessitate innovative approaches for sustainable and resilient aquaculture. Probiotics represent a promising avenue for mitigating the adverse effects of climate change on shellfish by promoting health, enhancing resilience, and contributing to the overall sustainability of this vital industry. As research and technology continue to advance, the integration of probiotics into shellfish farming practices may become a key strategy in adapting to the evolving environmental conditions.

Research endeavors have been focused on comprehending the complex interplay and mechanisms between environmental factors and the gut microbiome of aquatic organisms, including fish and shellfish. Studies have highlighted that external factors, including temperature, salinity, pH, chemical oxygen demand, total nitrogen, phosphorus, carbon, and inorganic nitrogen, influence the composition of the gut microbiome [84,85]. These factors can potentially increase disease prevalence within ecosystem habitats.

As a result, comprehending the influences of host microbiota interactions and cultivable organisms holds promise for enhancing predictions of biodiversity responses to climate change.

So far, most of the works on this topic have focused on possible scenarios and predictions when growing shellfish in conditions of climate change and on potential solutions to overcome them, and there is a rather small amount of research dealing with specific examples of shellfish. Among the research are studies from Čanak et al. [12] and Kovačić et al. [11], which found that queen scallop (*Aequipecten opercularis*) tolerates conditions of climate change during supplemented feeding with *Lpb. plantarum* I, LAB previously isolated from the above-mentioned bivalve. These studies confirmed that the use of indigenous LAB through feeding can improve the health status and survival of queen scallops in simulated climate change conditions.

## 6. Conclusions and Further Perspective

Climate change poses significant challenges to the mariculture industry, affecting water temperatures, precipitation patterns, sea-level rise, and more. However, the industry is actively responding to these challenges through various adaptation and mitigation strategies. By embracing climate-resilient species, improving infrastructure, utilizing monitoring systems, and promoting sustainable practices, aquaculture can navigate the changing climate landscape while continuing to provide a sustainable and reliable source of seafood.

The use of probiotics in shellfish farming in CC-OA conditions can be a useful strategy for improving the resistance and health of shellfish and optimizing production. Research has demonstrated that probiotics can exert beneficial effects on the digestive system, immune system, and stress resistance of shellfish, mitigating challenges like temperature and pH fluctuations. Moreover, probiotics have shown potential in reducing the adverse impacts of pathogenic microorganisms and enhancing the quality and growth of shellfish. However, further investigation is necessary to delve deeper into our understanding of the mechanisms behind probiotic action and their distinct effects on various shellfish species.

Additionally, optimal application strategies need to be developed to align with evolving growing conditions and the impacts of climate change.

Some of the potential suggestions that can help scientists and the industry, as well as small producers, are listed below:

- Develop formulations of probiotic cultures and models of treatment of fresh shellfish with probiotic cultures, with the aim of ensuring microbiological safety and extended shelf life;
- Develop models of packaging and preservation of shellfish;
- Investigate shellfish treated in this way will be accepted by consumers;
- Conduct scientific research on the positive impact of indigenous probiotic cultures on the microbiological safety and extended durability of shellfish;
- Educate small producers about the advantages and benefits of using bioprotective microbial cultures to improve the health and prolong the shelf life of shellfish;
- Develop ways of feeding certain types of bivalves depending on whether the breeding is carried out in aquariums or cages in the sea;
- Study the interaction of probiotic cultures with other microorganisms in the sea and the possible potential damage to the ecological system and food chain;
- Encourage breeders to add indigenous probiotic cultures, in a form available to the shellfish, during the feeding process.

In summary, a holistic approach that combines environmental, economic, and socio-political factors is imperative for accurately assessing and mitigating the impacts of CC-OA on mollusk mariculture at local and national scales.

## Data Availability

The original contributions presented in the study are included in the article, further inquiries can be directed to the corresponding author.

## References

[1] Mugwanya M., Dawood M.A., Kimera F., Sewilam H. (2022). Anthropogenic temperature fluctuations and their effect on aquaculture: A comprehensive review. Aquac. Fish..

[2] Dawood M.A., Noreldin A.E., Sewilam H. (2021). Long term salinity disrupts the hepatic function, intestinal health, and gills antioxidative status in Nile tilapia stressed with hypoxia. Ecotoxicol. Environ. Saf..

[3] Fazio F. (2019). Fish hematology analysis as an important tool of aquaculture: A review. Aquaculture.

[4] Ruby P., Ahilan B. (2018). An overview of climate change impact in fisheries and aquaculture. Clim. Chang..

[5] Weatherdon L.V., Magnan A.K., Rogers A.D., Sumaila U.R., Cheung W.W. (2016). Observed and projected impacts of climate change on marine fisheries, aquaculture, coastal tourism, and human health: An update. Front. Mar. Sci..

[6] Maulu S., Hasimuna O.J., Haambiya L.H., Monde C., Musuka C.G., Makorwa T.H., Munganga B.P., Phiri K.J., Nsekanabo J.D. (2021). Climate change effects on aquaculture production: Sustainability implications, mitigation, and adaptations. Front. Sustain. Food Syst..

[7] Gubbins M., Bricknell I., Service M. (2013). Impacts of climate change on aquaculture. MCCIP Sci. Rev..

[8] Frost M., Baxter J.M., Buckley P.J., Cox M., Dye S.R., Withers Harvey N. (2012). Impacts of climate change on fish, fisheries and aquaculture. Aquat. Conserv. Mar. Freshw. Ecosyst..

[9] Collins C., Bresnan E., Brown L., Falconer L., Guilder J., Jones L., Kennerley A., Malham S., Murray A., Stanley M. (2020). Impacts of Climate Change on Aquaculture. (MCCIP Science Review; No. 2020).

[10] Lebel L., Navy H., Jutagate T., Akester M.J., Sturm L., Lebel P., Lebel B. (2021). Innovation, practice, and adaptation to climate in the aquaculture sector. Rev. Fish. Sci. Aquac..

[11] Kovačić I., Žunec A., Matešković M., Burić P., Iveša N., Štifanić M., Frece J. (2023). Commercial quality, biological indices and biochemical composition of queen scallop *Aequipecten opercularis* in culture. Fishes.

[12] Čanak I., Kovačić I., Žunec A., Jakopović Ž., Kostelac D., Markov K., Štifanić M., Burić P., Iveša N., Frece J. (2023). Study of the impact of *Lactiplantibacillus plantarum* I on the health status of queen scallop *Aequipecten opercularis*. Appl. Sci..

[13] Zhao Y., Wang Y., Song Z., Shan C., Zhu R., Liu F. (2016). Development of a simple, low-cost and eurytopic medium based on *Pleurotus eryngii* for lactic acid bacteria. AMB Express.

[14] Šušković J., Kos B., Beganović J., Leboš Pavunc A., Habjanič K., Matošić S. (2010). Antimicrobial activity–the most important property of probiotic and starter lactic acid bacteria. Food Technol. Biotechnol..

[15] Wu C., Huang J., Zhou R. (2017). Genomics of lactic acid bacteria: Current status and potential applications. Crit. Rev. Microbiol..

[16] Kathiresan K., Thiruneelakandan G. (2008). Prospects of lactic acid bacteria of marine origin. Indian J. Biotechnol..

[17] Sica M.G., Olivera N.L., Brugnoni L.I., Marucci P.L., López-Cazorla A.C., Cubitto M.A. (2010). Isolation, identification and antimicrobial activity of lactic acid bacteria from the Bahía Blanca Estuary. Rev. Biol. Mar. Y Oceanogr..

[18] Muñoz-Atienza E., Gómez-Sala B., Araújo C., Campanero C., Del Campo R., Hernández P.E., Herranz C., Cintas L.M. (2013). Antimicrobial activity, antibiotic susceptibility and virulence factors of lactic acid bacteria of aquatic origin intended for use as probiotics in aquaculture. BMC Microbiol..

[19] Čanak I., Markov K., Gavrilović A., Bosanac P., Dujaković J., Jakopović Ž. (2018). Mikrobiološki i kemijski parametri ribe i školjkaša. Croat. J. Food Technol. Biotechnol. Nutr..

[20] Čanak I., Markov K., Jakopović Ž., Kostelac D., Čolak S., Mejdandžić D., Mattea Ž., Damir J., Frece J. In vitro characterization of *Lactobacillus plantarum* O1 isolated from gut of sea bream (*Sparus aurata*) as potential fish probiotic. Proceedings of the 4th International Conference of Food and Biosystems Engineering (FaBE 2019).

[21] Merrifield D.L., Balcázar J.L., Daniels C., Zhou Z., Carnevali O., Sun Y.Z., Hoseinifar S.H., Ringø E. (2014). Indigenous lactic acid bacteria in fish and crustaceans. Aquac. Nutr. Gut Health Probiotics Prebiotics.

[22] Pinto A.L., Fernandes M., Pinto C., Albano H., Castilho F., Teixeira P. (2009). Characterization of anti-Listeria bacteriocins isolated from shellfish: Potential antimicrobials to control non-fermented seafood. Int. J. Food Microbiol..

[23] Pavlova B.K., Ibryamova S.F., Arhangelova N.N., Doichev D.D., Ivanov R.I., Chipev N.H., Natchev N.D., Ignatova-Ivanova T.V. (2020). Integrative investigation on the ecology of the black sea mussel *Mytilus galloprovincialis* Lam. and its habitat. Ecol. Balk..

[24] Lee H.I., Hee Kim M., Young K.K., So J.S. (2010). Screening and selection of stress resistant *Lactobacillus* spp. isolated from the marine oyster (*Crassostrea gigas*). Anaerobe.

[25] Fajardo P., Atanassova M., Garrido-Maestu A., Wortner-Smith T., Cotterill J., Cabado A.G. (2014). Bacteria isolated from shellfish digestive gland with antipathogenic activity as candidates to increase the efficiency of shellfish depuration process. Food Control.

[26] Alonso S., Carmen Castro M., Berdasco M., de la Banda I.G., Moreno-Ventas X., de Rojas A.H. (2019). Isolation and partial characterization of lactic acid bacteria from the gut microbiota of marine fishes for potential application as probiotics in aquaculture. Probiotics Antimicrob. Proteins.

[27] Cunningham M., Azcarate-Peril M.A., Barnard A., Benoit V., Grimaldi R., Guyonnet D., Holscher H.D., Hunter K., Manurung S., Obis D. (2021). Shaping the future of probiotics and prebiotics. Trends Microbiol..

[28] Latif A., Shehzad A., Niazi S., Zahid A., Ashraf W., Iqbal M.W., Rehman A., Riaz T., Aadil R.M., Khan I.M. (2023). Probiotics: Mechanism of action, health benefits and their application in food industries. Front. Microbiol..

[29] Gaggìa F., Mattarelli P., Biavati B. (2010). Probiotics and prebiotics in animal feeding for safe food production. Int. J. Food Microbiol..

[30] Ringø E. (2020). Probiotics in shellfish aquaculture. Aquac. Fish..

[31] Gómez-Sala B., Muñoz-Atienza E., Sánchez J., Basanta A., Herranz C., Hernández P.E., Cintas L.M. (2015). Bacteriocin production by lactic acid bacteria isolated from fish, seafood and fish products. Eur. Food Res. Technol..

[32] Yang Q., Lü Y., Zhang M., Gong Y., Li Z., Tran N.T., He Y., Zhu C., Lu Y., Zhang Y. (2019). Lactic acid bacteria, *Enterococcus faecalis* Y17 and *Pediococcus pentosaceus* G11, improved growth performance, and immunity of mud crab (*Scylla paramamosain*). Fish Shellfish. Immunol..

[33] Labh S.N., Shakya S.R. (2014). Application of immunostimulants as an alternative to vaccines for health management in aquaculture. Int. J. Fish. Aquat. Stud..

[34] Elston R.A., Ford S.E. (2011). Shellfish diseases and health management. Shellfish Aquaculture and the Environment.

[35] Carnegie R.B., Arzul I., Bushek D. (2016). Managing marine mollusc diseases in the context of regional and international comerce: Policy issues and emerging concerns. Philos. Trans. R. Soc. B Biol. Sci..

[36] Aguilar-Macías O.L., Ojeda-Ramírez J.J., Campa-Córdova A.I., Saucedo P.E. (2010). Evaluation of natural and commercial probiotics for improving growth and survival of the pearl oyster, *Pinctada mazatlanica*, during late hatchery and early field culturing. J. World Aquac. Soc..

[37] Campa-Córdova A.I., González-Ocampo H., Luna-González A., Mazón-Suástegui J.M., Ascencio F. (2009). Growth, survival, and superoxide dismutase activity in juvenile *Crassostrea corteziensis* (Hertlein, 1951) treated with probiotics. Hidrobiológica.

[38] Sohn S., Lundgren K.M., Tammi K., Karim M., Smolowitz R., Nelson D.R., Rowley D.C., Gómez-Chiarri M. (2016). Probiotic strains for disease management in hatchery larviculture of the eastern oyster *Crassostrea virginica*. J. Shellfish. Res..

[39] Diep D.B., Nes I.F. (2002). Ribosomally synthesized antibacterial peptides in Gram positive bacteria. Curr. Drug Targets.

[40] Ringø E., Schillinger U., Holzapfel W., Mosenthin R., Zentek J., Żebrowska T. (2005). Antimicrobial activity of lactic acid bacteria isolated from aquatic animals and the use of lactic acid bacteria in aquaculture. Biology of Growing Animals.

[41] Sumon T.A., Hussain M.A., Sumon M.A.A., Jang W.J., Abellan F.G., Sharifuzzaman S.M., Brown C.L., Lee E.-W., Kim C.-H., Hasan M.T. (2022). Functionality and prophylactic role of probiotics in shellfish aquaculture. Aquac. Rep..

[42] Gao X., Zhang M., Li X., Han Y., Wu F., Liu Y. (2018). The effects of feeding *Lactobacillus pentosus* on growth, immunity, and disease resistance in *Haliotis discus hannai* Ino. Fish Shellfish. Immunol..

[43] Hadi J.A., Gutierrez N., Alfaro A.C., Roberts R.D. (2014). Use of probiotic bacteria to improve growth and survivability of farmed New Zealand abalone (*Haliotis iris*). N. Z. J. Mar. Freshw. Res..

[44] Grandiosa R., Mérien F., Young T., Van Nguyen T., Gutierrez N., Kitundu E., Alfaro A.C. (2018). Multi-strain probiotics enhance immune responsiveness and alters metabolic profiles in the New Zealand black-footed abalone (*Haliotis iris*). Fish Shellfish. Immunol..

[45] Grandiosa R., Young T., Van Nguyen T., Mérien F., Alfaro A.C. (2020). Immune response in probiotic-fed New Zealand black-footed abalone (*Haliotis iris*) under *Vibrio splendidus* challenge. Fish Shellfish. Immunol..

[46] Karim M., Zhao W., Rowley D., Nelson D., Gomez-Chiarri M. (2013). Probiotic strains for shellfish aquaculture: Protection of eastern oyster, *Crassostrea virginica*, larvae and juveniles againsl bacterial challenge. J. Shellfish. Res..

[47] El-Dakar A.Y., Shalaby S.M., Saoud I.P. (2007). Assessing the use of a dietary probiotic/prebiotic as an enhancer of spinefoot rabbitfish *Siganus rivulatus* survival and growth. Aquac. Nutr..

[48] Muller J.A., Ross R.P., Fitzgerald G.F., Stanton C. (2009). Manufacture of probiotic bacteria. Prebiotics and Probiotics Science and Technology.

[49] Martínez-Cruz P., Ibáñez A.L., Monroy Hermosillo O.A., Ramírez Saad H.C. (2012). Use of probiotics in aquaculture. Int. Sch. Res. Not..

[50] Lara-Flores M., Olvera-Novoa M.A., Guzmán-Méndez B.E., López-Madrid W. (2003). Use of the bacteria *Streptococcus faecium* and *Lactobacillus acidophilus*, and the yeast *Saccharomyces cerevisiae* as growth promoters in Nile tilapia (*Oreochromis niloticus*). Aquaculture.

[51] Ghosh S., Sinha A., Sahu C. (2008). Dietary probiotic supplementation in growth and health of live-bearing ornamental fishes. Aquac. Nutr..

[52] Dharmaraj S., Dhevendaran K. (2010). Evaluation of *Streptomyces* as a probiotic feed for the growth of ornamental fish *Xiphophorus helleri*. Food Technol. Biotechnol..

[53] Macey B.M., Coyne V.E. (2005). Improved growth rate and disease resistance in farmed *Haliotis midae* through probiotic treatment. Aquaculture.

[54] Balcázar J.L., Decamp O., Vendrell D., De Blas I., Ruiz-Zarzuela I. (2006). Health and nutritional properties of probiotics in fish and shellfish. Microb. Ecol. Health Dis..

[55] El-Haroun E.R., Goda A.S., Kabir Chowdhury M.A. (2006). Effect of dietary probiotic Biogen^®^ supplementation as a growth promoter on growth performance and feed utilization of Nile tilapia *Oreochromis niloticus* (L.). Aquac. Res..

[56] Lin H.Z., Guo Z., Yang Y., Zheng W., Li Z.J. (2004). Effect of dietary probiotics on apparent digestibility coefficients of nutrients of white shrimp *Litopenaeus vannamei* Boone. Aquac. Res..

[57] Chai P.C., Song X.L., Chen G.F., Xu H., Huang J. (2016). Dietary supplementation of probiotic Bacillus PC465 isolated from the gut of *Fenneropenaeus chinensis* improves the health status and resistance of *Litopenaeus vannamei* against white spot syndrome virus. Fish Shellfish. Immunol..

[58] Zhao J., Ling Y., Zhang R., Ke C., Hong G. (2018). Effects of dietary supplementation of probiotics on growth, immune responses, and gut microbiome of the abalone *Haliotis diversicolor*. Aquaculture.

[59] Lalloo R., Ramchuran S., Ramduth D., Görgens J., Gardiner N. (2007). Isolation and selection of *Bacillus* spp. as potential biological agents for enhancement of water quality in culture of ornamental fish. J. Appl. Microbiol..

[60] Wang Y.B., Xu Z.R., Xia M.S. (2005). The effectiveness of commercial probiotics in northern white shrimp *Penaeus vannamei* ponds. Fish. Sci..

[61] Dawood M.A.O., Koshio S., Ishikawa M., Yokoyama S. (2015). Effects of heat killed *Lactobacillus plantarum* (LP20) supplemental diets on growth performance, stress resistance and immune response of red sea bream, *Pagrus major*. Aquaculture.

[62] Dawood M.A.O., Koshio S., Ishikawa M., Yokoyama S. (2015). Effects of partial substitution of fish meal by soybean meal with or without heat-killed *Lactobacillus plantarum* (LP20) on growth performance, digestibility, and immune response of amberjack, *Seriola dumerili* Juveniles. BioMed Res. Int..

[63] Castex M., Lemaire P., Wabete N., Chim L. (2010). Effect of probiotic *Pediococcus acidilactici* on antioxidant defences and oxidative stress of *Litopenaeus stylirostris* under *Vibrio nigripulchritudo* challenge. Fish Shellfish. Immunol..

[64] Zheng X., Duan Y., Dong H., Zhang J. (2017). Effects of dietary *Lactobacillus plantarum* in different treatments on growth performance and immune gene expression of white shrimp *Litopenaeus vannamei* under normal condition and stress of acute low salinity. Fish Shellfish. Immunol..

[65] Rengpipat S., Rukpratanporn S., Piyatiratitivorakul S., Menasaveta P. (2000). Immunity enhancement in black tiger shrimp (*Penaeus monodon*) by a probiont bacterium (*Bacillus* S11). Aquaculture.

[66] Li H.D., Tian X.L., Dong S.L. (2019). Growth performance, non-specific immunity, intestinal histology and disease resistance of *Litopenaeus vannamei* fed on a diet supplemented with live cells of *Clostridium butyricum*. Aquaculture.

[67] Miao S., Han B., Zhao C., Hu J., Zhu J., Zhang X., Sun L. (2020). Effects of dietary *Pediococcus acidilactici* GY2 single or combined with *Saccharomyces cerevisiae* or/and β-glucan on the growth, innate immunity response and disease resistance of *Macrobrachium rosenbergii*. Fish Shellfish. Immunol..

[68] Amoah K., Huang Q.C., Dong X.H., Tan B.P., Zhang S., Chi S.Y., Yang Q.-H., Liu H.-Y., Yang Y.Z. (2020). *Paenibacillus polymyxa* improves the growth, immune and antioxidant activity, intestinal health, and disease resistance in *Litopenaeus vannamei* challenged with *Vibrio parahaemolyticus*. Aquaculture.

[69] Dash G., Raman R.P., Prasad K.P., Makesh M., Pradeep M.A., Sen S. (2015). Evaluation of paraprobiotic applicability of *Lactobacillus plantarum* in improving the immune response and disease protection in giant freshwater prawn, *Macrobrachium rosenbergii* (de Man, 1879). Fish Shellfish. Immunol..

[70] Kolanchinathan P., Kumari P.R., Gnanam T.S., John G., Balasundaram A. (2017). Research article performance evaluation of two probiotic species, on the growth, body composition and immune expression in *Penaeus monodon*. J. Fish. Aquat. Sci..

[71] Tepaamorndech S., Chantarasakha K., Kingcha Y., Chaiyapechara S., Phromson M., Sriariyanun M., Kirschke C.P., Huang L., Visessanguan W. (2019). Effects of Bacillus aryabhattai TBRC8450 on vibriosis resistance and immune enhancement in Pacific white shrimp, *Litopenaeus vannamei*. Fish Shellfish. Immunol..

[72] Li H., Tian X., Zhao K., Jiang W., Dong S. (2019). Effect of *Clostridium butyricum* in different forms on growth performance, disease resistance, expression of genes involved in immune responses and mTOR signaling pathway of *Litopenaeus vannamai*. Fish Shellfish. Immunol..

[73] Wu H.J., Sun L.B., Li C.B., Li Z.Z., Zhang Z., Wen X.B., Hu Z., Zhang Y.-L., Li S.K. (2014). Enhancement of the immune response and protection against *Vibrio parahaemolyticus* by indigenous probiotic *Bacillus strains* in mud crab (*Scylla paramamosain*). Fish Shellfish. Immunol..

[74] Sánchez-Ortiz A.C., Angulo C., Luna-González A., Álvarez-Ruiz P., Mazón-Suástegui J.M., Campa-Córdova Á.I. (2016). Effect of mixed-*Bacillus* spp isolated from pustulose ark *Anadara tuberculosa* on growth, survival, viral prevalence and immune-related gene expression in shrimp *Litopenaeus vannamei*. Fish Shellfish. Immunol..

[75] Duan Y., Wang Y., Dong H., Ding X., Liu Q., Li H., Zhang J., Xiong D. (2018). Changes in the intestine microbial, digestive, and immune-related genes of *Litopenaeus vannamei* in response to dietary probiotic *Clostridium butyricum* supplementation. Front. Microbiol..

[76] Hao K., Liu J.Y., Ling F., Liu X.L., Lu L., Xia L., Wang G.X. (2014). Effects of dietary administration of *Shewanella haliotis* D4, *Bacillus cereus* D7 and *Aeromonas bivalvium* D15, single or combined, on the growth, innate immunity and disease resistance of shrimp, *Litopenaeus vannamei*. Aquaculture.

[77] Interaminense J.A., Vogeley J.L., Gouveia C.K., Portela R.S., Oliveira J.P., Silva S.M., Coimbra M.R.M., Peixoto S.M., Soares R.B., Buarque D.S. (2019). Effects of dietary *Bacillus subtilis* and *Shewanella* algae in expression profile of immune-related genes from hemolymph of *Litopenaeus vannamei* challenged with *Vibrio parahaemolyticus*. Fish Shellfish. Immunol..

[78] Thiyagarajan V., Ko G.W.K. (2012). Larval growth response of the Portuguese oyster (*Crassostrea angulata*) to multiple climate change stressors. Aquaculture.

[79] Reid G.K., Gurney-Smith H.J., Marcogliese D.J., Knowler D., Benfey T., Garber A.F., Forster I., Chopin T., Brewer-Dalton K., Moccia R. (2019). Climate change and aquaculture: Considering biological response and resources. Aquac. Environ. Interact..

[80] Barton A., Waldbusser G.G., Feely R.A., Weisberg S.B., Newton J.A., Hales B., Cudd S., Eudeline B., Langdon C.J., Jefferds I. (2015). Impacts of coastal acidification on the Pacific Northwest shellfish industry and adaptation strategies implemented in response. Oceanography.

[81] Buck B.H., Nevejan N., Wille M., Chambers M.D., Chopin T., Buck B.H., Langan R. (2017). Offshore and multi-use aquaculture with extractive species: Seaweeds and bivalves. Aquaculture Perspective of Multi-Use Sites in the Open Ocean: The Untapped Potential for Marine Resources in the Anthropocene.

[82] Hai N.V. (2015). The use of probiotics in aquaculture. J. Appl. Microbiol..

[83] Liu L., Zhang S., Zhang D., Bi X. (2017). Control of harmful blue-green algae in ponds by berberine compound and probiotics. Fish. Sci..

[84] Sun F., Wang Y., Wang C., Zhang L., Tu K., Zheng Z. (2019). Insights into the intestinal microbiota of several aquatic organisms and association with the surrounding environment. Aquaculture.

[85] Sehnal L., Brammer-Robbins E., Wormington A.M., Blaha L., Bisesi J., Larkin I., Martyniuk C.J., Simonin M., Adamovsky O. (2021). Microbiome composition and function in aquatic vertebrates: Small organisms making big impacts on aquatic animal health. Front. Microbiol..

